# Development and Evaluation of Orthographic Knowledge Awareness Scale for Children Aged 6–12 Years

**DOI:** 10.3389/fpsyg.2022.874891

**Published:** 2022-07-12

**Authors:** Yachun Xie, Qu Xu, Liying Liu, Mengmeng Yao, Panting Liu, Meiling Tong, Qin Hong, Xia Chi

**Affiliations:** ^1^Department of Child Health, Women's Hospital of Nanjing Medical University (Nanjing Maternity and Child Health Care Hospital), Nanjing, China; ^2^School of Pediatrics, Nanjing Medical University, Nanjing, China

**Keywords:** Chinese characters, orthographic knowledge awareness, specific learning disabilities, scale, reliability, validity

## Abstract

**Objective:**

This study primarily aimed to develop an orthographic knowledge awareness scale in Mandarin for children aged 6–12 years. Related factors affecting orthographic knowledge awareness in children were analyzed, and a basis for individualized intervention was provided to improve reading and writing.

**Methods:**

A conceptual framework for orthographic knowledge awareness in children aged 6–12 years was determined through a detailed reading of the literature on Chinese character orthography, combined with qualitative interviews of the target population and consultation with experts. The orthographic knowledge awareness scale initially consisted of three versions: for grades 1–2 (210 items), grades 3–4 (207 items), and grades 5–6 (220 items), accumulating a total of 637 items. The initial scale was then used for the study involving children aged 6–12 years in Maanshan City, Jiangsu Province. Various approaches to screening items were comprehensively used to determine the formal version of the orthographic knowledge awareness scale. The official scale was ultimately used to conduct the third round of surveys among 1,354 children aged 6–12 years in ordinary primary schools located in 5 cities in Jiangsu Province, namely, Changzhou, Lianyungang, Nantong, Xuzhou, and Yangzhou. The reliability, validity, and discriminating power of the formal scale were evaluated.

**Results:**

A total of 360 items were included in the formal version of the orthographic knowledge awareness scale. The formal scale was divided into three versions for grades 1–2, 3–4, and 5–6. Each grade version consisted of 120 items. The scale was composed of the stroke awareness test, radical awareness test, and left–right reversal test. The cumulative variance contribution rates of grades 1–2, 3–4, and 5–6 were 82.47, 61.71, and 64.19%, respectively. The Cronbach's α coefficients of the three-grade version of the scale were 0.989, 0.946, and 0.938; the split-half reliability coefficients were 0.925, 0.766, and 0.847; and the test–retest reliability coefficients were 0.847, 0.895, and 0.8928, respectively.

**Conclusion:**

The proposed orthographic knowledge awareness scale for children aged 6–12 years exhibits good reliability and validity. The formal scale consisted of two dimensions: identification of left–right reversal at the stroke and radical levels and the left–right reversal at the whole character level. The two dimensions can more comprehensively reflect the ability of children to discriminate orthographic structures.

## Introduction

Children do not learn Chinese characters by rote memorizing each of them. Instead, they are aware of the orthographic aspects common to all characters (Bowers and Michita, 1998). Such awareness is helpful to their future learning of other characters (Lam and 林浩昌, 2006). Children also realize the relation between the radicals and the characters, i.e., semantic radicals provide a clue to the meanings of the characters, while phonetic radicals provide a clue to the sounds. Children have also gained orthographic knowledge about the way the characters should be composed, for example, some radicals can only appear at a certain position in the characters. Being aware of these general features of the characters should be helpful to the learning of other new characters in the future.

The physical attributes of modern Chinese characters can be classified into three: strokes, radicals, and whole characters. Orthographic knowledge refers to the azimuth structure of strokes or radicals in each Chinese character (Fan et al., 2018). Different strokes or radicals can only be combined in accordance with certain rules to form familiar Chinese characters. These standards are referred to as the rules of orthographic knowledge awareness (Chung et al., 2010; Yu-Lin et al., 2017). Radicals are used as an example. Awareness of radicals in Chinese characters means that children can recognize whether radicals actually exist in Chinese characters. Some radicals in Chinese characters, such as “口”, are located in different positions in the character. For instance, “口” can be found on the right side, at the top, on the left side, or at the bottom in the characters 扣, 吴, 味, and 吉. Some radicals can only appear in one position within the character. For instance, the radical “亻” can only be found on the left side of the Chinese characters 你, 他 and 什.

The study of orthographic knowledge awareness started in the Western phonetic language (English) region. However, the process of Chinese character recognition varies with the development of English literacy. Therefore, the research methods and conclusions of the orthographic knowledge of phonetic characters are not applicable to Chinese characters and cannot be used in China. Different from the linear arrangement of English scripts, Chinese characters are block characters, and each Chinese character is a tightly structured figure. Moreover, Chinese characters are ideographic characters with complex and changeable structures, and the structural defect of Chinese characters is the main feature of ideographic characters. A kind of linguistic knowledge that serves as a basis for processing Chinese characters is essential for awareness of Chinese character orthography.

Studies have shown that the 6–12 age interval is critical for the development of orthographic knowledge awareness in children (Ho et al., 2002; Chung et al., 2010), and such awareness is affected by age, reading ability, attention, and other related factors (Xiaochen et al., 2011; Yu-Lin et al., 2017) ^.^ As a basic cognitive ability of Chinese reading and spelling, orthographic knowledge awareness can help children advance from processing a single character to processing a series of characters (Law et al., 2018; Dong et al., 2019).

The lack of orthographic knowledge awareness is the main cause of impaired reading and writing in children with specific learning disabilities (Ho et al., 2002, 2004; Ho and Ma, 2010; Wang et al., 2014). Children with specific learning disabilities refer to children with normal intelligence but have one or more specific disorders in reading, writing, spelling, expression, calculation, and other aspects of basic mental processes. (Ho, 2010; Liu et al., 2014; Lishman, 2015). Special learning disabilities have an incidence rate of 5–18% (Hong, 2006; Qian et al., 2013; Chen et al., 2019). Such disorders can affect the reading, writing, and expression skills of individuals in school and daily life (Chan et al., 2007; Liu et al., 2016). Currently, no comprehensive and generalized tools exist to assess orthographic knowledge awareness in China. Therefore, an orthographic knowledge awareness scale for children aged 6–12 years needs to be developed based on the distinguishing characteristics of orthographic knowledge awareness in Chinese children. This scale has a clinical significance for the early identification and evaluation of children with specific learning disabilities and the determination of appropriate interventions for such disorders.

## Materials and Methods

### Participants

The inclusion criteria for the research subjects are as follows: (1) children aged 6–12 years who are in grades 1–6 of primary school; (2) agreement to participate in the survey. The exclusion criteria were as follows: (1) mentally retarded children. The “Combined Raven's Test” was used to evaluate the children's intelligence; children with IQ <70 were not included in the sample. (2) Children with hearing impairment, low vision, physical disability, or severe trauma. The parents and teachers of the children were surveyed to understand the children's hearing, vision, trauma, disease history, and learning situations. Children with hearing impairment, low vision, physical disability, or severe trauma were identified based on the medical records provided by the school clinics.

A total of 1,500 people were surveyed using the formal scale evaluation. We excluded 23 children with a history of neurological or psychiatric disorders. We further excluded 123 children who failed to submit their experimental data owing to data loss or refusal to attend the experiments. Ultimately, 1,354 valid questionnaires were obtained from the respondents consisting of 722 boys (53.3%) and 632 girls (46.7%). The boy-to-girl ratio was 1.14–1. The questionnaire distribution by grade was as follows: grades 1–2, 418 valid questionnaires (222 from boys and 196 from girls); grades 3–4, 473 valid questionnaires (249 from boys and 224 from girls); grades 5–6, 463 valid questionnaires (251 from boys and 212 from girls). The demographic characteristics of the subjects are listed in Table 1.

**Table 1:** Demographic characteristics of the subjects (*n* = 1,354).

**Characteristics**	**Grouping**	**Total number of people**	**Constituent ratio (%)**
Gender	Male	722	53.3
	Female	632	46.7
Grade	Grade 1–2	418	30.9
	Grade 3–4	473	34.9
	Grade 5–6	463	34.2
Occupation of parents	State Party and government organs, enterprises, and institutions in charge	85	6.3
	Professional and technical personnel	198	14.6
	Handle affairs personnel	84	6.2
	Service personnel	349	25.8
	Production personnel in agriculture, forestry, fishery, and profit industries; operators of production and transportation equipment; and servicemen	156	11.5
	Inconvenient classifier	298	22.0
	Unemployed	184	13.6
Educational level of parents	Junior high school and below	386	28.5
	High school and technical secondary school	634	46.8
	University and higher	334	24.7

### Construction of the Scale

#### Preparation of the First Draft of the Scale

First, the relevant literature was reviewed to analyze the assessment content and factors influencing Chinese character orthographic knowledge awareness. The connotation and extension of Chinese character orthographic knowledge awareness in children aged 6–12 years were defined by integrating qualitative interviews and expert consultation. Subsequently, the scale dimensions were preset, and an item pool with 850 items was established. A preliminary selection of items was conducted *via* group discussion on the target population and expert consultation to form a draft scale of 637 items. The scale consisted of stroke awareness testing, radical awareness testing, and left–right reversal testing. The scale was divided into three versions based on the grade level of the children: grades 1–2, 210 items; grades 3–4, 207 items; and grades 5–6, 220 items.

The scale includes three levels of structure: stroke, radical, and whole character. The first level examines stroke awareness, including stroke form error (bhA), stroke combination error (bhB), stroke increase (bhC), and stroke reduction (bhD). The second level measures radical awareness, including component addition (bjA), component reduction (bjB), change in component position (bjC), change in the ontological structure of a component (bjD), and assimilation error (bjE). The third level investigates whole-character awareness to distinguish left–right reversal, which includes left–right structural characters (zfA), up–down structural characters (zfB), and other structural characters (zfC) (upper-, middle-, and lower-structure characters, except for the left–right and up–down structures, semi-enclosed structures, single-character structures, and so on.). The details are listed in Table 2.

**Table 2:** Contents in the item pool of the orthographic knowledge scale for children aged 6–12 years.

**Contents of the scale**		**Number of scale entries for each level**			
		**Grades 1–2**	**Grades** **3–4**	**Grades 5–6**	**Demonstration**
Level of strokes	Incorrect stroke shape (bhA)	14	13	14	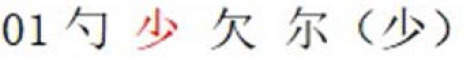
	Incorrect stroke combination relationship (bhB)	13	11	13	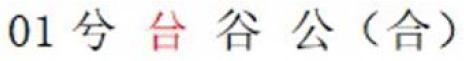
	Stroke increase (bhC)	18	19	17	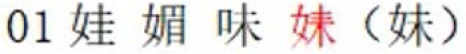
	Stroke reduction (bhD)	20	19	24	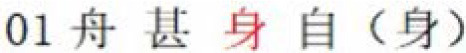
Level of radicals	Partial increase (bjA)	9	10	11	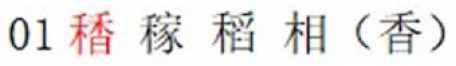
	Partial reduction (bjB)	9	9	10	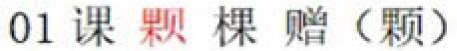
	Component position change (bjC)	17	18	18	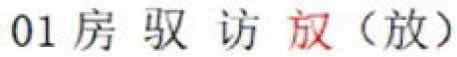
	Component body structure change (bjD)	33	32	32	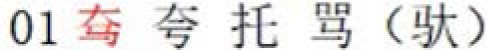
	Assimilation error (bjE)	10	11	10	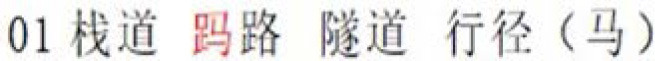
Level of whole characters	Left–right reversal structures (zfA)	27	27	29	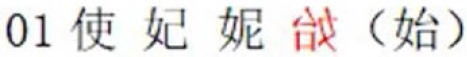
	Up–down structures (zfB)	16	16	20	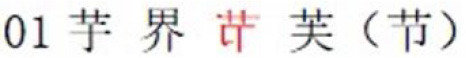
	Other structures (zfC)	24	22	22	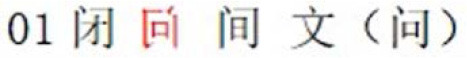
	Total entries	210	207	220	

*Examples of items are provided in Table 2, in which the chinese charaters marked in red are the incorrect forms of the target answer and the correct forms of the target answer are in parentheses*.

The items in the stroke awareness test were set based on the stroke content investigated. Each item was a multiple-choice question with four choices, including one inspected character (created non-characters) and three matching characters (true characters). The subjects were asked to select the non-characters. The characters selected for inspection were high-frequency characters. Among the inspected characters were basic and anamorphic strokes. Basic strokes included points, horizontal lines, vertical lines, apostrophes, lifts, folds, and hooks; anamorphic strokes are strokes formed when the basic strokes are in different positions or combined. Matching characters were selected from low-frequency characters in the basic Chinese character database used in primary schools. The criteria for selecting matching words were as follows: (1) similar to the character being examined, that is, identical or similar component/radical, identical or similar number of strokes, or identical or similar font structure, and (2) similar in pronunciation to the character being examined. In the radical awareness test, the items were set based on the radical content investigated. Each item was a multiple-choice question with four choices, including one inspected character (created non-characters) and three matching characters (true characters). The subjects were asked to select the non-characters. The characters selected for inspection were high-frequency characters. The inspected characters included radicals from modern common character radicals and name specifications. The position of radicals contains 22 common combinations of Chinese radicals. Matching characters were selected from the basic information base of Chinese characters used by primary school students; moreover, the criteria for selecting matching characters were identified with stroke awareness.

Items in the left–right reversal discrimination test were set in accordance with the whole-character content investigated. Each item is a multiple-choice question with four choices, including one inspected character (created non-characters) and three matching characters (true characters). The subjects were asked to select the non-characters. The items were set based on the whole character content investigated. The left–right reversal test has a mirror image relationship at the spatial level. For instance, “始” is reversed to “台女”. The selected characters (created left–right reversal) were high-frequency characters from the list of frequently used Chinese characters. These characters were in the Chinese curriculum standards for compulsory primary education formulated by the Ministry of Education. The characters for inspection were mainly left–right and top–bottom structures. The matching characters should be selected from the information base of Chinese characters used by primary school students, and the criteria for selecting matching characters were identified with stroke awareness.

#### Initial Scale Establishment

Convenience sampling was used to select an ordinary primary school in Maanshan City, Jiangsu Province. A total of 300 children met the inclusion criteria and thus were selected for pre-testing. On the basis of the survey data, different methods of screening items were used, producing three versions for grades 1–2, 3–4, and 5–6. Ultimately, 510 items were found in the scale; 170 items in each grade (including 50 items in the stroke), 65 items in the radical, and 55 items in the left–right reversal.

#### Formal Scale Formation

Random sampling was employed to select one ordinary primary school in Nantong City and another in Lianyungang City in Jiangsu Province. All these schools were of medium teaching level and scale in the city. The inclusion or exclusion of the research objects was determined based on the list of all children in grades 1–6 in primary schools and their basic information. Stratified sampling by grade and gender was subsequently performed; 25 boys and girls in each grade were selected using the random number table method, and 300 students in grades 1–6 were selected. A total of 600 students were thus selected from the 2 schools. The items were further screened based on the survey data, and initial reliability and validity tests were conducted to produce a formal scale with 360 items in 2 dimensions. The formal scale consisted of three levels: grades 1–2, 3–4, and 5–6, each of which had 120 items. The formal scale consisted of two dimensions: identification of left–right reversal at the stroke and radical levels (80 items) and the left–right reversal at the whole-character level (40 items).

#### Formal Scale Evaluation

Stratified random sampling was used to select 5 cities with different levels of economic development and geographic locations in Jiangsu Province: Changzhou, Lianyungang, Nantong, Xuzhou, and Yangzhou. From each urban area, a primary school with a medium teaching level and scale was randomly selected. From each grade, 25 boys and 25 girls were selected by using the random number table method. A total of 50 students from each grade comprised the 300 students selected from each school. Overall, 1,500 students were chosen from the five primary schools combined. From each school, 60 students were retested at intervals of 2 weeks, for a total of 300 students. The reliability, validity, and differentiation of the scale were evaluated based on the survey data.

#### Investigation and Quality Control

Before conducting the survey, the researchers explained the purpose and significance of the study to the parents and teachers of the students. Signed informed consent was then obtained from each student. Questionnaires containing questions on the basic living and learning conditions of the children were distributed to the parents and teachers of the children. The rules for filling out the questionnaire were explained in detail to the respondents. After the questionnaires were accomplished, the researcher collected the questionnaire and conducted logical checks and rechecks to ensure the integrity and accuracy of the data.

The investigators of the combined Raven test, orthographic knowledge awareness scale test, and literacy test were uniformly trained. They were familiar with the testing tool instructions and precautions. After filling in the questionnaire, the researcher collected the questionnaires and performed logical checks and rechecks to ensure the integrity and accuracy of the data. The investigators of the joint Raven test, Glabra, and literacy tests were trained uniformly. All testers were trained to familiarize themselves with the testing tool instructions and precautions. Questionnaires were distributed by the testers at the site. After completion, the questionnaires were checked and recovered in the test, and the omitted and erroneous parts of the questionnaire were promptly corrected.

### Literacy Assessment Scale

The scale included six versions for grades 1–6 (Xiaoling, 1996). The literacy scale for the first grade consisted of 10 groups, each consisting of about 25 common characters. The subjects were asked to pronounce all Chinese characters for no more than 30 min. Grades 2–6 had 10 literacy groups, with each group consisting of 6–33 common Chinese characters. The literacy scale for grades 2–6 adopted a collective test, requiring children to complete the questions by forming phrases or sentences for the targeted Chinese characters. The total literacy score for grades 1–2 was calculated by multiplying the number of correct groups according to their difficulty levels by different coefficients. The total literacy score for grades 3–6 was calculated by multiplying the number of correct answers in each group by different coefficients and adding the literacy base of the grade. The final result was the literacy of the child.

### Statistical Analysis

The following methods were comprehensively used for item selection: (1) descriptive analysis method: on the basis of the standard of difficulty coefficient (diff) > 0.6–0.7 and discriminative coefficient (disc) > 0.4, the items that failed to meet the difficulty and differentiation criteria were removed; (2) correlation coefficient method: the item with the correlation coefficient (*r*) <0.4 was the item to be deleted; (3) exploratory factor analysis method: the items with factor load value >0.4 and commonality >0.2 were kept; (4) Cronbach's α coefficient method: after the item was deleted, if the coefficient increased, the item was to be deleted; (5) critical ratio method: the total score of the items was divided into high and low groups—that is, the top 27% and the bottom 27%, respectively. The items with significant differences between high and low groups were retained (*P* < 0.05), and those without significant differences were eliminated. The principle underlying item deletion was that in the five aforementioned methods, if an item was to be deleted by the three methods simultaneously, the item had to be deleted. If an item was to be deleted by two or fewer methods, the item should be deleted or merged with professional knowledge. Content validity was evaluated by correlation analysis. Exploratory factor analysis was used to determine the factor structure, and confirmatory factor analysis was used to verify the structural validity of the scale. The reliability of the scale was evaluated using Cronbach's α coefficient, test–retest reliability coefficient, and split-half reliability coefficient. The statistical software SPSS 21.0 was used for statistical analysis. *T*-test or ANOVA was used to compare the differences in scores of different dimensions among populations with different characteristics. *P* < 0.05 was considered statistically significant.

## Results

### Scale Structure

**Figure 1:**
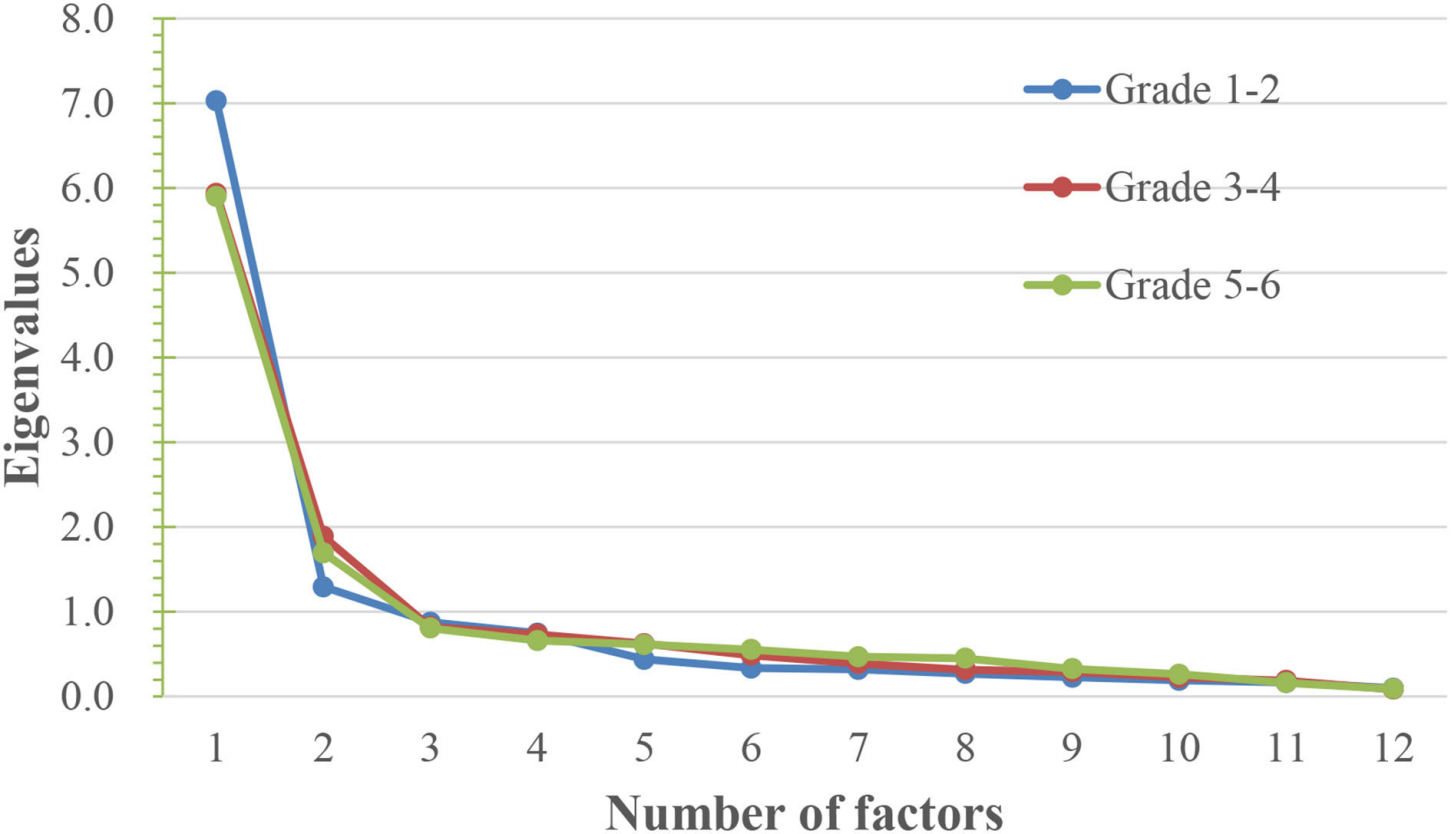
Gravel diagram of factor analysis.

Half of the survey data were randomly selected for exploratory factor analysis. The Kaiser–Meyer–Olkin (KMO) values of the three grade scales (grades 1–2, 3–4, and 5–6) were 0.936, 0.877, and 0.905, respectively, all of which were >0.60. The values of the Bartlett sphericity test were 5,422.97; 3,816.91; and 2,914.04, and their concomitant probabilities were < 0.05, meeting the factor analysis conditions. Principal component analysis was adopted for exploratory factor analysis. After the factor was rotated by the maximum variance, factors with characteristic values >1 were extracted. Two common factors were extracted for all scales, suggesting that the first two factors should be extracted, as shown in Figure 1. The number of factors was reset to 2 for factor analysis and the load of all items in the three grade scales exceeded 0.5. The results are listed in Tables 3–5. Grade 1–2 edition: The variance contribution rates of the two factors were 50.30 and 32.17, respectively, and the cumulative contribution rate was 82.47%. Grade 3–4 edition: The variance contribution rates of the two factors were 37.63 and 24.08, respectively, and the cumulative contribution rate was 61.71%. Grades 5–6 edition: The variance contribution rates of the two factors were 338.21 and 25.9, respectively, and the cumulative contribution rate was 64.19%.

**Table 3:** Exploratory factor analysis of each factor loading matrix for grades 1–2 scale (120 items).

	**Factor 1**		**Factor 2**		
**Items**	***N* of items**	**Loadings**	**Items**	***N* of items**	**Loadings**
bjC	17	0.865	zfC	10	0.921
bjB	10	0.845	zfB	10	0.889
bhC	5	0.828	zfA	20	0.886
bjA	11	0.824			
bhD	5	0.808			
bjE	12	0.773			
bhA	6	0.753			
bhB	7	0.736			
bjC	7	0.697			

**Table 4:** Exploratory factor analysis of each factor loading matrix for grades 3–4 scale (120 items).

	**Factor 1**		**Factor 2**		
**Items**	***N* of items**	**Loadings**	**Items**	**Items**	***N* of items**
bjC	12	0.831	zfC	21	0.947
bjB	17	0.819	zfB	8	0.924
bhC	10	0.793	zfA	11	0.854
bjA	11	0.785			
bhD	8	0.717			
bjE	5	0.713			
bhA	6	0.636			
bhB	6	0.534			
bjC	5	0.489			

**Table 5:** Exploratory factor analysis of each factor loading matrix for grades 5–6 scale (120 items).

	**Factor 1**		**Factor 2**		
**Items**	***N* of items**	**Loadings**	**Items**	**Items**	***N* of items**
bjC	17	0.783	zfC	20	0.937
bjB	14	0.769	zfB	9	0.933
bhC	7	0.723	zfA	11	0.908
bjA	6	0.713			
bhD	7	0.708			
bjE	10	0.676			
bhA	9	0.674			
bhB	6	0.633			
bjC	4	0.630			

### Factor Naming

Literature analysis was conducted. The items with high load values were summarized, and the two factors were identified as stroke and radical awareness test (F1, 9 items) and left–right reversal test (F2, 3 items).

### Reliability Test

The total Cronbach's alpha coefficient for the three grade scales was 0.938–0.989, and the Cronbach's alpha coefficient of each dimension ranged from 0.901 to 0.982. The split-half reliability coefficients of the scales for the three grades ranged from 0.766 to 0.925, and each dimension was between 0.887 and 0.976. The test–retest reliability coefficients of the three grade scales ranged from 0.847 to 0.928, and the dimensions were between 0.835 and 0.946, indicating that the scale exhibited high reliability. The details are listed in Table 6.

**Table 6:** Orthographic knowledge scale for children aged 6–12 years and various dimensional coefficients.

**Dimension**	**Grades 1–2**			**Grades 3–4**			**Grades 5–6**		
	**A**	**B**	**C**	**A**	**B**	**C**	**A**	**B**	**C**
F1 (Stroke and radical awareness test)	0.982	0.968	0.835	0.934	0.887	0.875	0.919	0.900	0.898
F2 (Left–right reversal test)	0.981	0.976	0.858	0.901	0.902	0.901	0.957	0.964	0.946
Total	0.989	0.925	0.847	0.946	0.766	0.895	0.938	0.847	0.928

*A: Cronbach's alpha coefficient; B: Split-half reliability coefficient; C: Test–retest reliability factor*.

### Validity Test

The Pearson correlation coefficients between the total scores of the three grade scales and the literacy scores of the children were 0.796, 0.801, and 0.764. The Pearson correlation coefficients of all dimensions ranged from 0.745 to 0.828. Correlation analysis was conducted between the scores of the scale at all levels and the Chinese academic performance of the children. The Pearson correlation coefficients between the total scores of the scale at all levels and the Chinese academic performance were 0.544, 0.562, and 0.576. The Pearson correlation coefficients of all dimensions ranged from 0.523 to 0.597. Correlation analysis was conducted between the scale score and the Chinese academic achievement of the children. The Pearson correlation coefficient between the total score of the three scales and Chinese academic achievement was 0.757–0.845. The Pearson correlation coefficients ranged from 0.751 to 0.858 for all dimensions. The aforementioned results indicate that the orthographic knowledge awareness scales exhibit good content validity.

The other half of the data were used to conduct a 2-factor model validation factor analysis for the 3 grade scales. The fitting results for the three grade scales were λ2/df = 0.308–1.053 <3. The goodness-of-fit index (GFI) ranged from 0.903 to 0.982, and the adjusted goodness-of fit-index (AGFI) ranged from 0.945 to 0.986. The comparative fit index (CFI) ranged from 0.912 to 0.975. The root–mean–square error of approximation varied from 0.001 to 0.046. All aforementioned results met the statistical requirements, indicating that the scale exhibited good structural validity.

### Reactivity Analysis

No statistically significant differences in the scores of all dimensions were found among children of different genders (*P* > 0.05). With regard to the stroke and radical awareness dimensions, the average scores of children of different ages showed statistically significant differences (*P* < 0.05). With regard to the left–right reversal test, the average scores were significantly different in other age groups, except for grades 3–4 and 5–6 (*P* < 0.05). Statistically significant differences in the average scores of stroke and radical awareness were noted among parents with different educational levels (*P* < 0.05). Similarly, statistically significant differences in the average scores of strokes and radical awareness were found between family monthly incomes (*P* < 0.05). The results are listed in Table 7.

**Table 7:** Comparison of average scores in various dimensions between children with different characteristics and their parents ($\bar{X}$ ± s).

**Variable**	**Stroke and radical awareness test**	**Left–right reversal test**
**Gender**		
Male	81.29 ± 15.75	37.91 ± 5.18
Female	79.98 ± 17.69	37.60 ± 5.44
**Grade**		
Grade 1–2	57.17 ± 30.81	39.75 ± 17.42
Grade 3–4	80.60 ± 16.80^a^	51.24 ± 7.15^a^
Grade 5–6	88.08 ± 14.40^a^	52.96 ± 5.91^a^
**Vocabulary**		
Difference	47.35 ± 11.41	35.35 ± 8.59
Poor	47.58 ± 11.59	36.43 ± 6.62
General	54.86 ± 12.59^b^	38.15 ± 4.19
Better	57.31 ± 14.27^b^	38.75 ± 5.69
It is good	65.32 ± 10.02^b^	39.27 ± 2.38
**Attention**		
Difference	44.84 ± 13.08	36.03 ± 7.36
Poor	49.19 ± 13.32^c^	37.00 ± 6.15
General	56.45 ± 11.21^c^	38.01 ± 4.21
Better	59.95 ± 12.36^c^	38.57 ± 4.78
It is good	65.04 ± 10.59 ^c^	38.84 ± 4.37
**Reading ability**		
Difference	43.00 ± 10.70	34.82 ± 9.02
Poor	47.97 ± 12.20^d^	36.33 ± 6.62
General	54.21 ± 12.14^d^	37.98 ± 4.30
Better	57.15 ± 14.03^d^	38.15 ± 5.20
It is good	66.61 ± 7.54^d^	39.22 ± 2.43
**Educational level of parents**		
Junior high school and below	51.04 ± 14.99	37.03 ± 6.28
High school/technical secondary school	56.76 ± 12.72^e^	37.99 ± 4.99
University and higher	62.43 ± 9.29^e^	38.06 ± 5.37
**Family monthly income (Yuan)**		
<5,000	52.18 ± 13.61	37.61 ± 4.72
5,000–10,000	53.67 ± 14.47	37.61 ± 5.65
10,000–30,000	57.77 ± 12.39^f^	37.88 ± 5.03
>30,000	58.50 ± 10.67^f^	38.19 ± 5.85

a *Compared with grades 1–2, P < 0.05*.

b *Compared with children's poor vocabulary, P < 0.05*.

c *Compared with children's poor attention span, P < 0.05*.

d *Compared with children's poor reading ability, P < 0.0.5*.

e *Compared with junior high school and below, P < 0.05*.

f *Compared with 5,000 Yuan or less, P < 0.05*.

## Discussion

In learning English words, the importance of phonological awareness has been well established, i.e., the “c” in the spelling “cat” signifies the /k/ sound in /kæt/. Much influenced by this, most research on learning Chinese characters investigates whether children realize what a radical signifies in a character. For example, the radical 木 (tree) provides a clue to the meaning of the character 椰 (coconut) (referred to as a part-whole relation in this dissertation). But, it was soon found that most children actually do not have much trouble recognizing this part-whole relation probably because 木 on its own is a very familiar character. Thus, to learn what a radical signifies in Chinese is much easier than the abstract task of phoneme segmentation in English. So what is important to learning Chinese characters?

A deficiency in orthographic knowledge awareness is the main cause of impaired reading and writing expression in Chinese children (Ho et al., 2003). Research on orthographic knowledge awareness in Western countries started earlier; however, the process of Chinese character recognition varies from the development of English literacy (Ho and Ma, 2010; Lin et al., 2020). English has the corresponding principle of phonics. For example, “read” can be divided into three phonemes “r-ea-d”. From the pronunciation of “read”, children can memorize the letters that make up words. However, Chinese characters are monosyllabic; one word corresponds to one sound, which does not conform to the principle of phonetic correspondence. The structure of Chinese characters is more complex, and the research methods and conclusions of the orthographic knowledge awareness of phonetic characters are not applicable to Chinese characters and cannot be applied in China.

Currently, Hong Kong, and Taiwan have conducted more in-depth research on orthographic knowledge. However, simplified and traditional characters largely vary in orthographic knowledge rules. Traditional characters have more complicated stroke characteristics and radical rules, and the positions of the radicals vary from those of simplified characters. The study of orthographic knowledge awareness in Hong Kong and Taiwan is not completely applicable to that in Mainland China. At present, comprehensive and universal evaluation tools for distinguishing and evaluating orthographic knowledge are lacking. Therefore, actively conducting research on orthographic knowledge awareness in children is highly necessary, which can provide a theoretical basis for the development of evaluation tools for orthographic knowledge awareness and the screening and diagnosis of Chinese dyslexic children.

In the current study, the Cronbach's α coefficients of the three-grade versions and each factor were higher than 0.901, the half-fold reliability exceeded 0.766, and the retest reliability was above 0.835. These results indicate that the scales exhibit good reliability. The KMO values of the three grade scales were higher than 0.8. The results of the exploratory factor analysis showed that the Bartlett sphericity test *P* < 0.001 and two common factors were extracted to explain the total variation of more than 60%, and the load of each factor exceeded 0.5. The results of confirmatory factor analysis showed that the fitting index of the three grade scales showed the goodness of fit of the model. The correlation validity between the total score and the literacy of the three grade scales exceeded 0.764. All aforementioned results indicate that the scale had a satisfactory validity. This study verified the reliability, validity, and stability of the scales. All indexes were in accordance with the psychometrics requirements and the model fit well. Therefore, the proposed scale can be used to evaluate orthographic knowledge awareness.

Studies have confirmed that the proposed scale, referred to as Children's Orthographic Knowledge Awareness Scale in this study, is closely related to the gender, age, reading, vocabulary comprehension, attention, educational level of parents, and family income of the children (Tong et al., 2009). The results of this study showed that no statistically significant difference was found between genders in the development of orthographic knowledge awareness in children aged 6–12, which was consistent with the literature (Liu et al., 2016). We found that orthographic knowledge awareness in children was associated with age. As children matured, the average score in stroke recognition and radical awareness increased. The recognition of left–right reversal is stronger in grades 1–2, but the ability to discriminate is not significantly different from that of children in grades 3–4 and 5–6, and these findings are consistent with other studies (Pyle et al., 2017). Reading ability, attention, and vocabulary can affect the ability of children to recognize character structures, which is consistent with the results of domestic and foreign research (Wu et al., 2012; Hui and Wang, 2016).

In summary, the Orthographic Knowledge Awareness Scale for children aged 6–12 years compiled in this study shows good reliability and validity. The two extracted dimensions can more comprehensively reflect the discrimination of orthographic knowledge awareness of children aged 6–12 years. However, the representativeness of the sample is limited because of the complex factors influencing the orthographic knowledge awareness and ability of the children, and this study was limited only to the Jiangsu Province. To ensure the stability and universality of the scale, the popularity of the test is further expanded, and the tests are conducted on subjects in different regions and from different races to ultimately develop a universally applicable orthographic knowledge awareness scale for children. The research and evaluation of influencing factors provide reliable measurement tools to promote the development of related studies on orthographic knowledge awareness in Chinese children.

## Conclusion

The proposed orthographic knowledge awareness scales for children aged 6–12 years exhibit good reliability and validity. The two dimensions can more comprehensively reflect the ability of children to discriminate orthographic structures.

## Data Availability Statement

The raw data supporting the conclusions of this article will be made available by the authors, without undue reservation.

## Ethics Statement

The studies involving human participants were reviewed and approved by Ethics Committee of Women's Hospital of Nanjing Medical University. Written informed consent to participate in this study was provided by the participants' legal guardian/next of kin. Written informed consent was obtained from the individual(s), and minor(s)' legal guardian/next of kin, for the publication of any potentially identifiable images or data included in this article.

## Author Contributions

XC and QH developed the idea for the study. LL, MY, and PL collected the data. YX and QX did the analyses. MT contributed reagents/materials/analysis tools. YX wrote the manuscript. All authors contributed to the article and approved the submitted version.

## Funding

This work was supported by Grants from National Natural Science Foundation of China (8167359), Sub-projects of the National Key R&D Program (2016YFC1000204-6), Jiangsu Province Science and Education Strengthening Health Medicine Innovation Team (CXTDA2017001), Six Talent Peaks High-Level Talents in Jiangsu Province (WSN-165), and the Nanjing Technological Development Program (zkx18044).

## Conflict of Interest

The authors declare that the research was conducted in the absence of any commercial or financial relationships that could be construed as a potential conflict of interest.

## Publisher's Note

All claims expressed in this article are solely those of the authors and do not necessarily represent those of their affiliated organizations, or those of the publisher, the editors and the reviewers. Any product that may be evaluated in this article, or claim that may be made by its manufacturer, is not guaranteed or endorsed by the publisher.
